# Urine-Derived Stem Cells: Challenges in Isolation, Biological Identity, and Therapeutic Potential in CKD-Associated Fibrosis

**DOI:** 10.3390/ijms27157038

**Published:** 2026-08-05

**Authors:** Queenesa Amabel Sunjaya, Ahmad Faried, Rudi Supriyadi, Jonny Jonny, Hiqmah Yusi Yana

**Affiliations:** 1 Faculty of Medicine, Universitas Padjadjaran, Jatinangor 45363, Indonesia; ahmad.faried@unpad.ac.id (A.F.); rudi.supriyadi@unpad.ac.id (R.S.); 2 Department of Neurosurgery, Faculty of Medicine, Universitas Padjajaran—RS UNPAD University Hospital, Jatinangor 45363, Indonesia; 3 Department of Internal Medicine, Faculty of Medicine, Universitas Padjadjaran, Bandung 40161, Indonesia; 4 Faculty of Military Medicine, Indonesia Defense University, Bogor 16810, Indonesia; 5 Department of Internal Medicine, Nephrology Division, Gatot Soebroto Central Army Hospital, Jakarta 10410, Indonesia; hiqmahyusi@gmail.com

**Keywords:** urine-derived stem cells, chronic kidney disease, renal fibrosis, regenerative medicine, renal progenitor cells, paracrine signaling

## Abstract

Urine-derived stem cells (UDSCs) have emerged as a promising cell source for regenerative medicine due to their non-invasive procurement, high proliferative capacity, and potential relevance to kidney-specific repair. Unlike conventional mesenchymal stem cells (MSCs) obtained from bone marrow or adipose tissue, UDSCs originate from multiple regions of the urinary tract and exhibit a unique biological profile that combines MSC characteristics with features of renal progenitor populations. This review provides a comprehensive overview of the current understanding of UDSC biology, including their origin, isolation strategies, morphology, immunophenotypic characteristics, differentiation potential, and secretory profile. Particular attention is given to the expression of renal lineage-associated markers and pluripotency-related factors that may contribute to their regenerative capacity. The bioactive mediators of UDSCs regulate inflammation, oxidative stress, angiogenesis, and extracellular matrix remodeling, thereby influencing key pathways implicated in chronic kidney disease (CKD)-associated fibrosis. Furthermore, the intrinsic renal progenitor signature of UDSCs may provide advantages in renal homing and tissue-specific repair compared with conventional MSC populations. Despite encouraging preclinical findings, significant challenges remain, including cellular heterogeneity, inconsistent isolation efficiency, lack of standardized characterization criteria, and limited clinical validation. Collectively, current evidence positions UDSCs as a biologically distinct and therapeutically attractive platform for kidney regeneration.

## 1. Introduction

Accumulating clinical research evidence indicates that mesenchymal stem cells (MSCs) are generally safe and may provide therapeutic benefits in both acute and chronic kidney diseases [1]. A Previous in vivo study reported that adipose-derived mesenchymal stem cells (AD-MSCs) differentiated into renal tubular epithelial cells in models of acute kidney injury, suggesting a potential role in tubular regeneration [2]. Another in vitro study demonstrated that MSCs differentiated into renal epithelial lineage when co-cultured with injured renal cells [3]. The differentiation of MSCs into renal and urinary lineages is recognized as a promising avenue for kidney repair. However, preclinical studies demonstrating consistent and functionally integrated differentiation of MSCs into mature renal tubular cells remain limited [3,4]. Therefore, it is increasingly proposed that the renoprotective effects of MSCs are primarily mediated through paracrine mechanisms, whereby secreted cytokines, growth factors, and extracellular vesicles modulate inflammation, reduce apoptosis, and stimulate endogenous repair processes rather than directly replacing damaged tubular cells [5].

Due to the paracrine-mediated effects of MSCs, there has been a growing interest in UDSCs as a unique and accessible source that may possess MSC-like characteristics while providing specific benefits for kidney-related regenerative purposes. Zhang et al. first talked about UDSCs in 2008. They showed that human urine has a variety of cells that can grow and change into different types of cells. In their initial characterization, three primary cellular categories were delineated: fully differentiated cells, cells in the process of differentiation, and progenitor-like cells [6]. Phenotypically, subsets of UDSCs were found to express several surface markers commonly associated with MSCs, including CD90, CD73, and CD105 [7]. The overlap in marker expression prompted subsequent investigations into whether a fraction of these cells might exhibit MSC-like properties. Since the initial report, multiple studies have explored the biological characteristics of UDSCs, examining their proliferative capacity, immunophenotypic profile, and differentiation potential [8]. One of the frequently highlighted advantages of UDSCs is their reported propensity to differentiate toward renal lineage cells, suggesting possible relevance in kidney-specific regenerative applications [9]. In addition, their non-invasive mode of isolation represents a practical advantage over traditional MSC sources such as bone marrow or adipose tissue [10].

The identification of UDSCs has generated growing interest within the field of regenerative medicine, particularly in urology and nephrology. A preclinical study has described regenerative potential in vitro, including lineage-specific marker expression and endothelial, osteogenic, chondrogenic, adipogenic, skeletal myogenic and neurogenic lineage differentiation [8]. In vivo investigations using animal models have reported improvements in selected histological and functional parameters following UDSC administration [11]. Preliminary clinical explorations in CKD have also been described [12]. However, the available evidence is limited by small sample sizes, early-phase study designs, and a lack of long-term follow-up, preventing definitive conclusions regarding clinical efficacy and safety. Despite these encouraging observations, the overall body of evidence remains relatively early-stage and heterogeneous in quality.

However, the regenerative claims surrounding UDSCs need to be interpreted with caution, as most of the available evidence is derived from small studies with short follow-up and limited mechanistic validation. A universally accepted definition of their biological identity has not yet been established and variability in marker expression suggests that UDSCs may constitute a heterogeneous population that remains inadequately characterized [8]. Such methodological inconsistencies make it difficult to compare across studies, and careful considerations need to be taken for translational application, especially with regard to sterility, genetic stability, reproducibility and long-term safety. In this regard, the present review discusses the biological identity of UDSCs and the mechanistic basis of their anti-fibrotic effects in CKD. This review integrates current preclinical and nascent clinical evidence to highlight the potential contributions of the unique renal progenitor features and paracrine functions of UDSCs in attenuating renal fibrosis and identifies the key biological and translational challenges to be addressed before clinical translation.

## 2. Literature Search Strategy

This narrative review was conducted through a literature search of PubMed, Scopus, ScienceDirect, and other relevant reference lists. Google Scholar was additionally searched using the following keywords: “urine-derived stem cells”, “urine-derived mesenchymal stem cells”, “chronic kidney disease”, “kidney fibrosis”, and “renal regeneration”. Additional relevant studies were identified through manual screening of reference lists from eligible articles. Publications were selected based on their relevance to the biological identity, isolation, characterization, molecular mechanism, and therapeutic applications of UDSC in CKD. No formal systematic review protocol or meta-analysis methodology was applied.

## 3. Urine-Derived Stem Cells’ Procurement

### 3.1. Origin of UDSC

Urine is not merely a passive waste product but a dynamic and chemically complex biofluid that reflects the body’s metabolic state. It contains a high concentration of waste solutes, including urea, salts, and organic acids, and exhibits variable pH and osmotic pressure [13]. The cells that line the urinary tract are always under stress because of the changing and difficult chemical environment. This very harsh environment is what makes it necessary for the kidney, ureters, and bladder to have a high rate of cell regeneration and turnover [14]. Consequently, the urinary tract is populated by regional stem and progenitor cells responsible for tissue maintenance and repair [15]. Some of these regenerative cells are naturally shed and can be non-invasively harvested from voided urine, where they are known as UDSCs [6].

UDSCs are not a single, homogeneous population but originate from various specific locations along the urinary tract [8]. They can be broadly categorized based on their origin in the upper versus the lower urinary tract. The Table 1 below breaks down these specific origins, compiled from [6,8,9,16,17,18,19,20,21,22,23,24,25,26,27,28,29].

The specific original source of a UDSC (e.g., whether it came from the renal tubule or the bladder urothelium) significantly influences its behavior in the laboratory. Their origin affects cellular morphology in vitro, optimal culture requirements, phenotypic characteristics (including surface markers), and differentiation potential [8,30]. Rather than representing a single cell type, UDSCs comprise a heterogeneous population of stem and progenitor cells originating from various regions of the urinary tract, ranging from the glomerulus to the bladder epithelium, all identified by their presence in urine [31].

### 3.2. Isolation and Culture of UDSCs

The isolation of UDSCs exploits their intrinsic ability to adhere to plastic culture surfaces and their robust proliferative capacity, which distinguishes them from terminally differentiated cells also present in urine [6]. This method is non-invasive, cost-effective, and reproducible, and generally involves centrifugation, washing, and subsequent culture in specialized media [17]. Following these steps, the cell pellet is resuspended in culture medium and plated [32]. Although the initial yield is relatively low, approximately 2–7 stem cells per 100 mL of urine, these cells demonstrate high clonogenic potential [6]. Initial colonies typically appear within 5–9 days, and the cells exhibit a doubling time of around 45 h, allowing expansion of a single clone to approximately 1.0 × 10^8^ cells within 6–7 weeks [6,33]. UDSCs also maintain a normal karyotype after multiple passages and express telomerase activity [6,34]. Telomerase is an enzyme that adds telomeric repeats to chromosome ends, counteracting telomere shortening during cell division and thus preventing replicative senescence. Telomerase activity is an important functional marker of self-renewal; it maintains chromosomal integrity and allows UDSCs to proliferate extensively over many passages without genomic instability, supporting their capacity for long-term expansion and differentiation [34]. Therefore, a single non-invasive urine sample can provide a sufficient number of stem cells for potential therapeutic applications without the need for surgical procedures.

However, isolating UDSCs from patients with CKD presents additional biological hurdles that can compromise consistency and therapeutic utility. Nephron loss and tubular atrophy reduce the number of progenitor-like cells shed into urine [35], while the uremic, inflammatory environment impairs cell viability, lowers proliferation rates, elevates reactive oxygen species and senescence markers, and compromises differentiation capacity–particularly in older or diabetic patients, making autologous therapy challenging [36,37]. The inherent yield of urinary stem cells is already low (success rates ranging from 10 to 73%, with only a few progenitor cells per 100 mL) [32]. In the context of CKD, this efficiency is drastically reduced. For instance, isolation success rates in patients with diabetic nephropathy drop significantly to 47.4%, compared to 86.7% in healthy individuals [37]. Moreover, CKD urine samples often contain proteinuria, hematuria, or bacterial contamination [38], which increase the risk of culture contamination and affect experimental conditions. Finally, the isolated cell population is still heterogeneous, with differentiated cells, apoptotic cells, and few progenitor cells, which raises problems for enrichment and data interpretation [32]. These challenges highlight the need for optimized, CKD-tailored isolation protocols to harness the full therapeutic potential of UDSCs in renal disease.

## 4. Biological Identity of UDSCs

**Figure 1:**
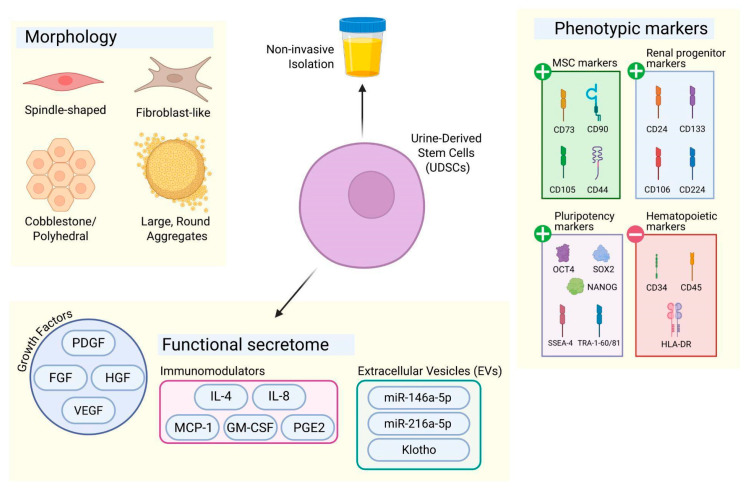
Biological characteristics of UDSCs. UDSCs are obtained through non-invasive urine collection and exhibit heterogeneous morphological phenotypes, including spindle-shaped, fibroblast-like, cobblestone/polyhedral, and large rounded aggregate-forming cells. Their immunophenotypic profile combines canonical MSC markers (CD73, CD90, CD105, and CD44) with renal progenitor markers (CD24, CD133, CD106, and CD224), while expressing selected pluripotency-associated markers (OCT4, SOX2, NANOG, SSEA-4, and TRA-1-60/81) and lacking hematopoietic markers (CD34, CD45, and HLA-DR). Functionally, UDSCs exert regenerative effects primarily through their secretome, including growth factors (vascular endothelial growth factor (VEGF), fibroblast growth factor (FGF), hepatocyte growth factor (HGF), and platelet-derived growth factor (PDGF)), immunomodulatory mediators (Interleukin-4 (IL-4), interleukin-8 (IL-8), monocyte chemoattractant protein-1 (MCP-1), granulocyte-macrophage colony-stimulating factor (GM-CSF), and prostaglandin E2 (PGE2)), and extracellular vesicles carrying bioactive cargo such as miR-146a-5p, miR-216a-5p, and Klotho. Biological characteristics of UDSCs. Created in BioRender (https://BioRender.com/wvwtwuj, (accessed on 25 July 2026)).

Despite these biological and logistical hurdles, successful isolation and expansion of UDSCs yield a cell population with a distinctive and reproducible biological identity. Once established in culture, these cells can be characterized by three fundamental pillars: their characteristic morphology, their surface marker profile, and their secreted metabolite byproducts. Understanding these features is essential not only for confirming successful isolation but also for predicting therapeutic behavior. The following sections therefore describe the morphological phenotypes of UDSCs, the expression of key surface markers that define their stemness and lineage potential, and the bioactive secretome that underpins their paracrine and anti-fibrotic functions (Figure 1).

### 4.1. Morphology

The initial step in harnessing the therapeutic potential of any stem cell population lies in its accurate identification and characterization. Before delving into complex genotypic or proteomic profiles, the first and most immediate clue to a cell’s identity is its morphology. The shape, size, and adherence patterns of cells in culture serve as a fundamental phenotypic marker, providing the initial evidence that a putative stem cell population has been successfully isolated [39]. For UDSCs, these visual characteristics are not merely descriptive; they are intrinsically linked to the cells’ biological state, stage of culture, and differentiation capacity [33]. Upon primary culture, UDSCs exhibit a distinct and characteristic morphological profile. They do not present as a single, uniform population but rather as a heterogeneous culture displaying specific, reproducible shapes. According to Culenova et al. [33] and Zhang et al. [6], freshly isolated UDSCs typically appear as small, adherent cells with a classic rice-grain or spindle-shaped morphology. As the culture expands, colonies often evolve, showing a mix of elongated, fibroblast-like cells and, in some instances, polyhedral or cobblestone-shaped cells indicative of epithelial lineage commitment [40]. In addition, certain populations develop as large, round cells that cluster together into dome-shaped colonies or spheroid-like aggregates, a feature linked to progenitor or undifferentiated stem cell states [41]. These aggregates, sometimes forming embryoid body-like structures, are considered early markers of clonogenic potential and highlight the strong regenerative capacity of UDSCs. This morphological dynamism is a key identifier, distinguishing UDSCs from other mesenchymal stem cell types and providing a real-time window into their health and differentiation potential.

#### 4.1.1. Rice Grain/Spindle-Shaped Morphology

UDSCs that look like rice grains or spindles are long and have tapered ends. This is a common shape in UDSC cultures [42] and is a sign of MSC-like populations. This morphology indicates mesenchymal identity, associated with differentiation into osteogenic, chondrogenic, myogenic, and adipogenic lineages [33]. Spindle-shaped UDSCs show robust proliferation, with colony doubling times of ~45–50 h, underscoring strong expansion potential [9]. This rapid growth and multipotency make them valuable for regenerative medicine where scalability and differentiation versatility are essential.

#### 4.1.2. Fibroblast-like Cells

UDSC often appear fibroblast-like: flat, elongated cells with a central oval nucleus and cytoplasmic extensions [43]. This morphology is common among cultured MSCs, reflecting adaptability and ease of expansion [39]. Similar to connective tissue fibroblasts, these cells produce extracellular matrix (ECM) components that contribute to structural support and tissue repair [42], highlighting their relevance in wound healing and tissue engineering applications [17]. Although sometimes grouped within the spindle-shaped category, fibroblast-like UDSCs tend to exhibit greater ECM production and stronger stromal support functions compared to typical spindle-shaped cells [33]. Despite these distinctions, they maintain multipotent capabilities, emphasizing their functional versatility in regenerative medicine [44].

#### 4.1.3. Polyhedral or Cobblestone-Shaped Cells

UDSCs may also exhibit an angular or polygonal morphology, forming flat cells that grow in tightly packed monolayers [33]. These cells often organize into a characteristic “cobblestone” pattern, which is typically associated with epithelial cell organization [45]. This phenotype is thought to originate from urothelial lining cells or renal tubular epithelial cells, giving them barrier-forming properties that are advantageous for applications such as urothelial tissue engineering and bladder reconstruction [17]. Despite their epithelial-like morphology, these cells continue to express MSC-associated surface markers, indicating that they retain stemness and multipotent potential. This combination of epithelial features and stem cell characteristics underscores their functional versatility in regenerative medicine [33].

#### 4.1.4. Large, Round with Cell Aggregation

Large, rounded UDSCs tend to aggregate into colonies or spheroid-like clusters, often forming dome-shaped structures or embryoid body–like formations [33]. The presence of dome structures is associated with highly active stem cells exhibiting strong regenerative potential, while embryoid body formation reflects early developmental processes and supports their stemness and differentiation ability [45]. Such aggregation is commonly observed in progenitor or undifferentiated stem cells, which display high proliferative capacity prior to spreading [46,47]. These colonies typically emerge within 3–9 days after plating and serve as indicators of clonogenic potential [33]. Therefore, rounded and aggregated UDSCs represent a reservoir of undifferentiated cells with strong expansion capacity and developmental plasticity, making them valuable for regenerative and developmental research.

As summarized in Table 2, the distinct morphological subpopulations of UDSCs directly reflect underlying differences in their molecular identities. The following section details the specific biomarker and phenotypic profiles that define these various UDSC subsets.

### 4.2. Biomarkers of UDSCs

UDSCs exhibit a range of MSC-associated surface markers, including CD73, CD90, CD29, CD44, CD146, CD105, CD166, CD54, STRO-1, VIM and CD117 [48,50]. Critically, UDSCs consistently display the three canonical antigens–CD73, CD90, and CD105–that, as established by the International Society for Cellular Therapy (ISCT), constitute the ‘minimal phenotypic fingerprint’ required to define cultured MSCs [49]. In addition to MSC-associated markers, UDSCs also express renal progenitor surface markers such as CD24, CD133 (Prominin-1), CD106, and CD224 [8]. These markers are critical for nephrogenesis during fetal kidney development and remain detectable in adult renal progenitor populations localized within proximal tubules (CD24^+^CD133^+^CD106^−^) and Bowman’s capsule (CD24^+^CD133^+^CD106^+^) [18,53,54]. The latter subset demonstrates the capacity to differentiate into diverse renal cell types, including podocytes and tubular epithelial cells. UDSCs also have surface markers that can help to identify their pluripotency, such as tumor rejection antigen-1-60 (TRA-1-60), tumor rejection antigen-1-81 (TRA-1-81), and stage-specific embryonic antigen-4 (SSEA-4). TRA-1-60 and TRA-1-81 represent glycosylated epitopes associated with podocalyxin and are widely recognized as reliable indicators of undifferentiated human embryonic stem cells. Similarly, SSEA-4, a glycosphingolipid expressed on the cell membrane, is routinely employed to distinguish pluripotent stem cells from differentiated progeny. UDSCs express CD140b, CD13, and CD146 [8,55,56], endothelial/vascular-associated markers, although CD140b’s expression is not that stable–can be negative or positive depending on the study [8,19,57]. Moreover, UDSCs were negative for the expression of CD34, CD45, CD31, HLA-DR, CD19, CD14, and CD11b [8,51,56]. The absence of CD34 and CD45 further delineates their MSC phenotype in accordance with ISCT standards [6,7,8]. Consequently, the presence of these renal lineage-associated markers in UDSCs suggests a stronger predisposition toward renal differentiation compared to conventional MSCs, potentially enhancing their utility in kidney regeneration strategies [18,53,54].

UDSCs express a range of pluripotency-associated markers, including SRY-box transcription factor 2 (SOX2), octamer-binding transcription factor 4 (OCT4), and NANOG, alongside the transcription factors *Kruppel-like factor 4 (KLF4)* and *MYC proto-oncogene (c-MYC)* [8,51]. Notably, while KLF4 and c-MYC are widely recognized for their roles in cellular reprogramming [52], they exhibit complex, context-dependent roles in tumorigenesis; *c-MYC* is a well-established oncogene, while *KLF4* can function as either a tumor suppressor or an oncogene depending on the cellular environment [58]. While pluripotency-associated markers such as OCT4, SOX2, and NANOG define embryonic stem cells and iPSCs [52], their expression in UDSCs is limited and context-dependent [51]. Native UDSCs exhibit a baseline MSC-like phenotype while containing a renal progenitor subpopulation that expresses a limited set of pluripotency-associated markers, with partial or inducible expression of pluripotency factors reported under specific culture or reprogramming conditions [59]. Compared to conventional MSCs, UDSCs exhibit a more robust pluripotent molecular signature, characterized by the expression of core pluripotency transcription factors and embryonic stem cell markers [51,60]. This highlights UDSCs as a flexible cell source that bridges somatic progenitor identity with potential reprogramming capacity.

UDSCs display a comprehensive panel of renal lineage-specific markers that collectively reflect their nephron progenitor origin and differentiated functional capabilities. Core transcriptional regulators such as SIX Homebox 2 (SIX2), CBP/P300-Interacting Transactivator with Glu/Asp-Rich C-Terminal Domain 1 (CITED1), Wils tumor 1 (WT1) [61,62,63,64], paired box gene 2 (PAX2) [65], and paired box gene 8 (PAX8) [64] are central to nephron progenitor identity, maintaining the cap mesenchyme during fetal kidney development and guiding lineage specification toward podocytes and tubular epithelia. Structural and lineage markers including Uroplakin Ia, cytokeratin 7, and cytokeratin 19 further delineate epithelial differentiation, reflecting the urothelial and tubular components of renal tissue [6,33]. Functional proteins such as aquaporins (AQP1, AQP2), uromodulin (UMOD), and the sodium-potassium-chloride cotransporter SLC12A1 define specialized transport properties of proximal and distal nephron segments, while the mineralocorticoid receptor NR3C2 underscores hormonal regulation of electrolyte balance [8,42]. Podocyte-specific markers such as nephrin (NPHS1) reinforce the glomerular lineage commitment of UDSCs, linking them to the filtration barrier architecture [42,54]. This composite framework suggests that UDSCs are not merely multipotent but exhibit a predisposition toward renal differentiation, thereby enhancing their relevance for kidney regeneration and disease modeling compared to conventional MSCs.

UDSCs express a panel that underscores their angiogenic potential. The native UDSC population exhibits an adhesive and pericyte-like signature, characterized by the expression of NG2 (CSPG4) [24,25] and E-cadherin (CDH1) [26,27]. The functional endothelial differentiation capacity of UDSCs has been rigorously demonstrated through the upregulation of several definitive vascular markers. Following induction with endothelial growth medium, UDSCs acquire a mature endothelial phenotype, marked by the induction of von Willebrand Factor (vWF), an essential glycoprotein mediating platelet adhesion and hemostasis, and endothelial Nitric Oxide Synthase (eNOS), a key enzyme responsible for nitric oxide production, which is critical for vasodilation and vascular homeostasis [17,19]. Furthermore, the expression of vascular endothelial growth factor (VEGF) receptors, namely Kinase Insert Domain Receptor (KDR/VEGFR-2) and Fms-like Tyrosine Kinase-1 (FLT-1/VEGFR-1), is significantly upregulated upon differentiation, enabling UDSCs to respond to angiogenic cues and participate in neovascularization [17]. The coordinated expression of these receptors and functional enzymes not only confirms endothelial lineage commitment but also equips UDSCs with the machinery to contribute to both large-vessel architecture and microvascular networks.

### 4.3. Metabolite Product of UDSC

UDSCs have a strong paracrine functionality, secreting important growth factors such as vascular endothelial growth factor (VEGF), platelet-derived growth factor (PDGF), fibroblast growth factor (FGF) and hepatocyte growth factor (HGF). VEGF is a master regulator of angiogenesis. It is also secreted endogenously by UDSCs. Under hypoxic conditions, VEGF can be amplified to promote capillary formation and cell survival [28,66]. PDGF, particularly the BB isoform, exerts direct effects on UDSC fate by actively promoting their neuronal differentiation [45]. FGF signaling plays a multifaceted role; FGF-1 is present in the UDSC secretome [67], while FGF2 (bFGF) has been identified as essential for maintaining the self-renewal of SIX2^+^ renal progenitor subsets via the TGFβ-SMAD2/3 pathway [59]. HGF, a potent stimulator of proliferation and tissue protection, is constitutively secreted by UDSCs and has been shown to enhance their secretion of small extracellular vesicles (sEVs), which are critical mediators of intercellular communication [59,68]. Consequently, this coordinated growth factor profile establishes UDSCs as potent paracrine effectors, enabling them to orchestrate angiogenesis, direct lineage specification, and modulate the local microenvironment.

Beyond soluble growth factors, a fundamental mechanism of stem cell paracrine signaling relies on extracellular vesicles (EVs), particularly exosomes. Before detailing the specific vesicular cargo of UDSCs, it is essential to contextualize their fundamental biology. Exosomes are nano-sized vesicles (30–150 nm in diameter) actively secreted by cells. Unlike passive cellular debris, they are formed via the endosomal pathway and deliberately loaded with a selective cargo of lipids, proteins, and nucleic acids—most notably microRNAs (miRNAs). Functionally, exosomes serve as highly stable intercellular messengers that protect their molecular payload from enzymatic degradation in biological fluids. In regenerative medicine, they offer distinct theoretical advantages over whole-cell transplantation: they cannot undergo uncontrolled proliferation (eliminating tumorigenicity risks), they lack MHC class II surface molecules (minimizing immunogenicity), and their small size allows them to cross biological barriers to deliver bioactive cargo directly to injured resident cells [69,70].

UDSCs leverage these EVs alongside soluble factors to exert profound immunomodulatory effects, which are critical for resolving the chronic inflammation that drives CKD fibrosis. This immunomodulatory capacity is dynamic and significantly amplified when UDSCs are “licensed” by an inflammatory microenvironment. Upon exposure to activated peripheral blood mononuclear cells (PBMNCs) or pro-inflammatory cytokines, UDSCs upregulate key immunoregulatory mediators, including IL-8, MCP-1, GM-CSF, CCL5 (RANTES), and CXCL1 (GROα) [17,33,71]. At the molecular level, UDSCs mediate immune crosstalk through distinct, targeted mechanisms. First, they drive lymphocyte suppression: through the secretion of CCL5 and GM-CSF, UDSCs induce cell-cycle arrest in activated T-lymphocytes, preventing their proliferation without triggering apoptosis, while concurrently downregulating pathogenic Th1 and Th17 effector cells [72]. Second, they orchestrate macrophage polarization; the secretion of interleukin-4 (IL-4) and prostaglandin E2 (PGE2) acts synergistically on resident macrophages. PGE2 binds to EP2 and EP4 G-protein coupled receptors on macrophages, triggering intracellular cyclic AMP (cAMP) signaling that shifts macrophages from a pro-inflammatory M1 state to an anti-inflammatory, pro-reparative M2 state. This M2 polarization is crucial for degrading excess fibrotic ECM [73]. Furthermore, TGF-β plays a context-dependent role in this network; beyond its well-known pro-fibrotic functions, it is a critical component of the FGF2-driven pathway that maintains the self-renewal of SIX2^+^ renal progenitor subsets within UDSCs [59].

Building upon this immunological foundation, UDSC-derived EVs possess a highly specialized cargo tailored for renal repair. While they share the structural lipid bilayer (rich in cholesterol, sphingomyelin, and phosphatidylserine) of typical exosomes, UDSC-EVs are uniquely enriched in specific miRNAs, proteins, and the anti-aging factor Klotho [29,74]. Among the most well-characterized miRNA cargo are miR-216a-5p and miR-146a-5p. miR-216a-5p targets phosphatase and tensin homolog (PTEN) to suppress tubular cell apoptosis via the Akt pathway, while miR-146a-5p downregulates Interleukin-1 receptor-associated kinase 1 (IRAK1) to inhibit NF-κB-driven inflammation, collectively mitigating ischemia/reperfusion injury [75,76]. Additionally, UDSC-derived exosomes specifically target and neutralize IL-1β-mediated pathways, rescuing local renal tissues from degeneration [77]. Notably, the delivery of Klotho—a potent anti-fibrotic protein whose decline drives CKD progression—directly restores endogenous Klotho loss in injured kidneys, enhances tubular cell proliferation, and reinforces local anti-fibrotic signaling [74].

In addition to their vesicular and cytokine secretomes, UDSCs mediate robust paracrine effects through lipid mediators, most notably PGE2 [78]. Beyond its mechanistic role in M2 macrophage polarization detailed above, PGE2 serves as a critical systemic effector molecule, orchestrating the overall downregulation of Th1/Th17 immune responses in a PGE2-dependent manner to reduce renal inflammation [79]. Crucially, PGE2 also exerts direct renoprotective effects by activating endogenous regenerative programs. Studies have demonstrated that PGE2 can activate Sox9^+^ renal progenitor cells via the Yap signaling pathway, promoting their differentiation into proximal tubular epithelial cells while actively suppressing fibrosis, thereby significantly improving kidney function following injury [80]. Consequently, this multifaceted paracrine profile—encompassing growth factors, targeted molecular immunomodulation, specialized EV cargo, and lipid mediators—positions UDSCs as uniquely equipped to reshape the fibrotic microenvironment of the diseased kidney.

## 5. Mechanistic Basis of UDSC-Mediated Fibrolysis in CKD

Chronic kidney disease (CKD) leads to fibrosis or sclerosis through a complex, self-sustaining pathomechanism initiated by persistent injury of renal parenchymal cells, including podocytes, tubular epithelial cells, and mesangial cells [81]. These injured cells release pro-inflammatory cytokines and chemokines, including MCP-1, TNF-α, and IL-6, which recruit monocytes, macrophages, and other immune cells into the kidney, amplifying local inflammation [82]. The inflammatory milieu activates pro-fibrotic signaling pathways, with TGF-β as the central mediator: it induces epithelial-to-mesenchymal transition (EMT) in tubular epithelial cells and podocytes, converting them into myofibroblasts that secrete ECM proteins such as collagen I, collagen III, and fibronectin [82,83,84]. Additional pathways, including angiotensin II from the renin-angiotensin system, connective tissue growth factor (CTGF), Wnt/β-catenin, and Notch signaling, reinforce fibroblast activation and ECM deposition [82,85,86]. Resident fibroblasts, pericytes, and circulating fibrocytes also differentiate into myofibroblasts, further expanding the population of matrix-producing cells [82,87]. As ECM accumulates, capillary rarefaction occurs, reducing microvascular density and oxygen delivery, which leads to hypoxia. Hypoxia itself stimulates HIF-1α and other signaling cascades that exacerbate apoptosis, inflammation, and fibrogenesis. Structurally, the kidney becomes progressively stiffened, with glomerulosclerosis and tubulointerstitial fibrosis disrupting nephron architecture, impairing filtration, and worsening proteinuria [88]. Proteinuria adds another layer of injury by stressing tubular epithelial cells and perpetuating inflammatory and fibrotic signaling [89]. Over time, this maladaptive repair process replaces functional renal parenchyma with scar tissue, leading to irreversible decline in glomerular filtration rate (GFR).

In CKD, glomerulosclerosis and tubulointerstitial fibrosis represent two distinct pathological processes, and each produces characteristic clinical manifestations [90]. Glomerulosclerosis primarily affects the glomeruli, the kidney’s filtration units, leading to impaired barrier function [90,91]. Clinically, this manifests as proteinuria (often nephrotic-range), hematuria, and progressive decline in GFR. Patients may develop edema, hypoalbuminemia, and hyperlipidemia as secondary consequences of protein loss, along with hypertension due to glomerular injury and maladaptive activation of the renin-angiotensin system [91,92]. By contrast, tubulointerstitial fibrosis involves scarring of the renal interstitium and tubular structures, producing symptoms more related to impaired tubular function [90]. This includes defective urine concentration (leading to polyuria and nocturia), electrolyte disturbances such as metabolic acidosis, sodium wasting, or potassium imbalance, and reduced responsiveness to hormones like erythropoietin, contributing to anemia of CKD. As fibrosis progresses, patients experience fatigue, bone mineral disorders, and uremic symptoms due to declining overall renal clearance [93].

**Figure 2:**
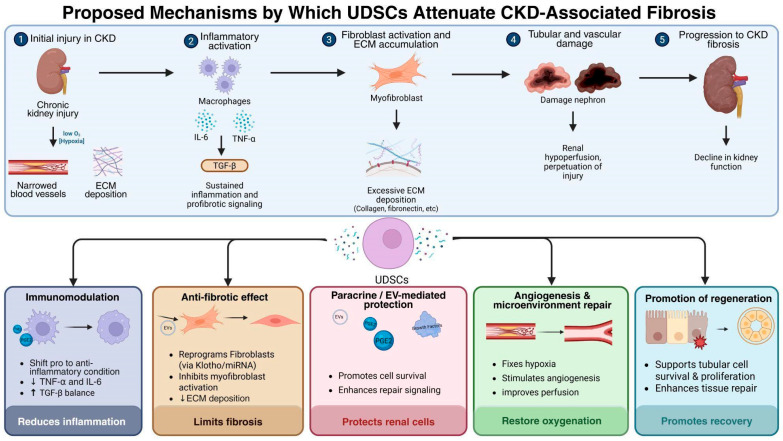
Proposed mechanisms by which UDSCs attenuate CKD-associated fibrosis. Persistent renal injury initiates inflammatory activation characterized by macrophage infiltration and increased production of pro-inflammatory cytokines, including TNF-α and IL-6, leading to TGF-β-mediated pro-fibrotic signaling. Sustained inflammation promotes myofibroblast activation and ECM deposition, resulting in tubular injury, renal hypoperfusion, progressive nephron damage, and ultimately CKD-associated fibrosis with declining renal function. UDSCs exert renoprotective effects primarily through paracrine signaling and EV-mediated mechanisms. Their secretome contains growth factors, PGE2, Klotho, and regulatory microRNAs that collectively modulate immune responses, suppress myofibroblast activation, inhibit ECM accumulation, promote angiogenesis, improve renal perfusion and oxygenation, enhance tubular cell survival and proliferation, and facilitate tissue repair. Collectively, these mechanisms contribute to attenuation of renal fibrosis and preservation of kidney structure and function. Created in BioRender (https://BioRender.com/4bc4757, (accessed on 25 July 2026)).

Fibrosis is central to CKD progression and therapeutic strategies are increasingly directed at targeting fibrolysis, the breakdown or reversal of fibrotic tissue, to restore homeostasis. In this respect, UDSCs are considered promising candidates since their biological identities, such as differentiation potential, paracrine signaling, secretome composition, and immunomodulatory capacity, directly intersect with the mechanisms governing fibroblast activity, extracellular matrix turnover and inflammatory resolution (Figure 2).

UDSCs exhibit significant differentiation potential, capable of generating various mesodermal lineages, including osteogenic, chondrogenic, and myogenic cells [10,94,95]. This highlights their importance in tissue repair and regenerative applications. This multipotency constitutes a fundamental aspect of their therapeutic potential; however, their anti-fibrotic effects are primarily facilitated through paracrine and secretory mechanisms rather than direct lineage replacement [96,97,98]. Beyond differentiation, UDSCs exhibit key biological characteristics that enhance their utility: they possess high proliferative capacity and clonogenicity, ensuring a sustained therapeutic presence [94,95]; they maintain genomic stability and adaptability, allowing long-term culture and safe application [95]; and they exert immunomodulatory effects by secreting cytokines and growth factors that dampen inflammation and regulate immune cell activity [10,72,95].

Effective renal homing is a prerequisite for stem cell therapy in CKD. The CD44-hyaluronic acid (HA) axis is the principal recruitment mechanism: injury upregulates HA in the kidney, creating a chemotactic gradient. CD44 binds HA, guiding cell migration. This is confirmed by anti-CD44 antibody blockade and by CD44 knockout MSCs failing to home or promote recovery, effects restored by wild-type CD44 but not a HA-binding mutant [99]. Both UDSCs and bone marrow stem cells (BMSCs) express CD44, UDSCs display consistently higher levels (often >99.5% positivity) [17,25], and BMSCs show variable or declining expression with passage [100]. This quantitative advantage translates into more robust homing to HA-rich renal injury sites. Additionally, NK cells exhibit lower cytotoxicity against UDSCs than BMSCs, conferring a survival advantage [17,72].

The most distinctive advantage of UDSCs is their intrinsic renal progenitor identity. Unlike BMSCs from non-renal tissues, UDSCs naturally originate from the kidney and retain a renal-specific transcriptional and surface-marker profile. Transcriptional profiling reveals nephrogenesis-associated factors (SIX2, CITED1, WT1, PAX2, PAX8), with SIX2 indicating epigenetic priming toward renal fate, a property MSCs lack [8,59]. Beyond transcription factors, UDSCs express surface markers CD24, CD133, CD106, and CD224 that are absent or negligible in MSCs. CD133^+^CD24^+^CD106^+^ cells differentiate into podocytes and tubular epithelia; CD133^+^CD24^+^CD106^−^ cells are tubular-committed progenitors resistant to apoptosis [8,18,20]. This compartmentalization is unique to renal progenitors. CD224 (GGT1) is a glutathione-metabolizing enzyme that neutralizes oxidative stress, a major fibrosis driver [8,83], providing intrinsic cytoprotection that MSCs lack. Consequently, UDSCs selectively localize to proximal tubules (CD106^−^) or Bowman’s capsule (CD106^+^), addressing both tubulointerstitial fibrosis and glomerulosclerosis with anatomical precision.

The renal-lineage specificity of UDSCs translates directly into enhanced anti-fibrotic activity in CKD. UDSCs exert significant anti-fibrotic activity through their secretome and metabolite byproducts, which collectively reshape the microenvironment of the diseased kidney [96]. UDSCs secrete growth factors (VEGF, HGF, FGF, and PDGF), cytokines/chemokines (IL-6, IL-8, MCP-1, and TGF-β), extracellular vesicles (carrying anti-fibrotic miRNAs, proteins, and Klotho mRNA), and PGE_2_. Growth factors counteract hypoxia, stimulate angiogenesis, and promote tissue remodeling, thereby suppressing the central pro-fibrotic driver TGF-β [101,102,103,104,105]. Cytokines and chemokines fine-tune immune responses, balancing leukocyte recruitment and inflammatory signaling to prevent excessive fibroblast activation [33,106,107,108,109,110]. Extracellular vesicles from UDSCs are particularly potent, delivering regulatory molecules that reprogram fibroblasts, suppress pro-fibrotic pathways, enhance ECM degradation and reinforce local Klotho protein expression in renal tubular cells [21,22,23,29,111,112]. Klotho is a potent anti-fibrotic factor whose decline drives CKD progression. Combining strong endogenous Klotho expression with renal progenitor markers gives UDSCs a synergistic advantage over MSCs, overcoming poor homing, inconsistent efficacy, and lack of kidney-specific bioactivity. Finally, UDSCs require no artificial differentiation protocols; they are epigenetically aligned with the kidney, unlike MSCs that need preconditioning or engineering. This intrinsic readiness yields more reliable anti-fibrotic outcomes in CKD models.

To fully appreciate the therapeutic utility of UDSCs in CKD, it is important to compare their biological identity with conventional stromal cell sources. Table 3 provides a comprehensive comparison of UDSCs against BM-MSCs and AD-MSCs, highlighting the intrinsic renal progenitor features and Klotho-rich secretome that give UDSCs a distinct mechanistic advantage in treating renal fibrosis.

## 6. Translational Bottleneck, Controversies, and the “CKD Paradox”

While the preclinical promise of UDSCs is undeniable, a critical evaluation of the literature reveals significant translational bottlenecks that current reviews often gloss over. To transition UDSCs from bench to bedside for CKD, the field must confront three major controversies: the “CKD Paradox” of autologous therapy, a fundamental identity crisis regarding characterization, and unresolved biosafety concerns.

The most pressing controversy is the “CKD Paradox.” The primary appeal of UDSCs lies in the feasibility of autologous transplantation—using the patient’s own cells to avoid immune rejection. However, the very patients who stand to benefit most from UDSC therapy (those with advanced CKD) present the most hostile biological environment for cell isolation. As discussed, nephron loss and tubular atrophy physically reduce the pool of shed progenitor cells [35], while the uremic, inflammatory milieu severely impairs the viability, proliferative capacity, and differentiation potential of the few cells that are shed [36,37]. This creates a frustrating clinical paradox: the sicker the patient, the harder it is to harvest therapeutically viable autologous UDSCs. This raises a contentious debate in the field—should researchers invest in optimizing complex ex vivo rescue protocols to rehabilitate a patient’s damaged autologous cells, or pivot toward allogeneic “off-the-shelf” UDSC banks from healthy donors, which introduces theoretical immunological risks despite the immune-privileged nature of MSCs?

Compounding this is a fundamental identity crisis. The ISCT established minimum criteria for MSCs based primarily on bone marrow-derived populations [49]. However, UDSCs frequently defy these conventional boundaries. They express the canonical MSC markers (CD73, CD90, CD105) but concurrently express embryonic stem cell markers (SSEA-4, TRA-1-60, TRA-1-81) and renal progenitor markers (CD24, CD133) that are entirely absent in standard MSCs [8,51]. Because UDSCs are a heterogeneous population originating from multiple anatomical niches along the urinary tract, different isolation protocols yield highly variable subpopulations [8,32]. The lack of UDSC-specific consensus criteria means that what one laboratory defines as a “UDSC” may be fundamentally different in phenotype and secretome from another’s, making cross-study comparisons unreliable and hindering regulatory approval.

Finally, the biosafety profile of UDSCs requires rigorous scrutiny. The expression of pluripotency-associated transcription factors (OCT4, NANOG, SOX2) alongside the well-established oncogene *c-MYC* [8,51,58] inevitably raises theoretical concerns regarding tumorigenicity. While current in vivo models of CKD have not reported teratoma formation or sarcomas following UDSC administration [8], the long-term oncogenic risk of injecting cells with an active c-MYC profile into a chronically inflamed, fibrotic renal microenvironment remains completely uncharted. Current clinical evidence is limited to early-phase, short-term safety trials [12]. Until robust, long-term post-transplantation surveillance data is available, the tumorigenic potential of UDSCs remains a significant translational worry that must be transparently addressed rather than dismissed.

## 7. Conclusions

Despite exhibiting morphological similarities to MSCs, the uniqueness of UDSCs lies primarily in their distinctive biomarker profile. UDSCs’ intrinsic expression of renal progenitor markers confers a kidney-specific identity. This lineage-primed state enables superior homing to injured nephron segments, enhanced paracrine secretion of renoprotective factors, and intrinsic antioxidant cytoprotection. Consequently, UDSCs overcome the issues of poor homing, inconsistent efficacy, and non-specific bioactivity that currently limit MSC-based therapies for CKD fibrosis. Nevertheless, their isolation–though non-invasive–remains technically demanding, especially in CKD patients. In CKD, nephron loss, uremic inflammation, contamination (proteinuria, hematuria, bacteria), and cellular heterogeneity further compromise isolation, yielding few genuine progenitors.

Finally, a major knowledge gap remains: the vast majority of studies characterizing UDSCs and testing their therapeutic efficacy have been conducted using cells derived from healthy young donors. Whether UDSCs isolated from patients with advanced CKD, diabetes, or other comorbidities retain the same proliferative capacity, marker expression profile, and anti-fibrotic potency remains largely unexplored. Future research must prioritize the isolation and functional assessment of UDSCs from diseased individuals, as well as the development of standardized, clinically compliant protocols for their expansion and delivery. Only then can the true therapeutic potential of UDSCs for CKD-associated fibrosis be fully realized.

## Figures and Tables

**Table 1:** Potential cellular sources of urine-derived stem cells along the urinary tract.

Anatomical Origin	Cell Type	Function	Interaction with UDSC
Upper urinary tract (renal)	Renal Tubular Cells	Reabsorption and secretion of solutes in the nephron tubules.	UDSCs contain CD133^+^CD24^+^CD106^−^ cells that act as tubular-committed progenitors resistant to apoptosis, and UDSCs exhibit a strong propensity to differentiate toward renal tubular epithelial lineages [8,9,18,20].
	Glomerular Pericytes	Surround capillaries in the glomerulus; regulate capillary stability.	The native UDSC population exhibits an adhesive and pericyte-like signature, characterized by the expression of NG2 (CSPG4) and E-cadherin (CDH1), allowing them to mimic pericyte support functions [24,25,26,27].
	Renal Interstitial Cells	Provide structural framework; mediate inflammation/fibrosis responses.	UDSC-derived extracellular vesicles deliver regulatory molecules that directly reprogram resident interstitial fibroblasts and suppress pro-fibrotic pathways (e.g., TGF-β), preventing them from becoming myofibroblasts [21,22,23,29].
	Vascular Endothelial Cells	Line blood vessels; regulate angiogenesis and vascular tone.	UDSCs endogenously secrete VEGF to promote capillary formation, and under induction, they express mature endothelial markers (vWF, eNOS, VEGFR-2) to actively participate in neovascularization [16,17,19,28].
Lower urinary tract	Epithelial (Urothelial) Cells	Line the internal surface of the ureter, urinary bladder, and urethra; provide barrier function.	UDSCs are primarily shed or isolated from this layer. While they originate here, UDSCs retain a primitive stem cell state rather than terminal urothelial differentiation [6,8].

**Table 2:** Morphological and phenotypic characteristics of distinct UDSC subpopulations in culture.

UDSC Population	Morphological Profile	Phenotypic Profile	Functional Significance
Rice grain/Spindle-shaped	Long cells with tapered ends; classic MSC-like appearance.	MSC Markers: CD73, CD90, CD105 [8,48,49].	Indicates strong mesenchymal identity. Shows robust proliferation (~45–50 h doubling time) and high capacity for osteogenic, chondrogenic, and adipogenic differentiation [8,33].
Fibroblast-like	Flat, elongated cells featuring a central oval nucleus and distinct cytoplasmic extensions.	MSC Markers + Stromal Markers: CD73, CD90, CD105, Vimentin (VIM) [48,50].	High production of extracellular matrix (ECM) components. Provides strong stromal support, making them highly valuable for wound healing and tissue engineering [17,33,42].
Polyhedral/Cobblestone-shaped	Angular or polygonal flat cells that grow in tightly packed monolayers.	MSC + Epithelial Markers: Cytokeratin 7, Cytokeratin 19, Uroplakin Ia, CD73/CD90/CD105 [8,33,42].	Originates from urothelial/tubular cells. Provides barrier-forming properties essential for urothelial tissue engineering and bladder reconstruction [17,33].
Large, Round with Aggregation	Large, rounded cells that cluster into dome-shaped colonies or embryoid body-like spheroids.	Pluripotency/Progenitor Markers: SSEA-4, TRA-1-60, TRA-1-81, OCT4, NANOG [48,51,52].	Represents a reservoir of undifferentiated cells. Indicates high clonogenic potential, strong regenerative capacity, and developmental plasticity [33,45,46].

**Table 3:** Comparative analysis of UDSCs, BM-MSCs, and AD-MSCs in the context of biological properties and anti-fibrotic therapeutic potential in CKD.

Feature/Parameter	UDSCs	BM-MSCs	AD-MSCs
Source	Voided urine (Non-invasive); cost-effective. Low initial yield but high clonogenic potential [3,21,22]	Bone marrow aspirate (highly invasive); painful; moderate yield [113].	Liposuction (minimally invasive); high initial yield [113].
Proliferative Capacity	Rapid doubling time (~45–50 h). Expresses telomerase activity, allowing long-term expansion without genomic instability [3,5,23,24].	Slower proliferation rate; tendency for senescence and reduced differentiation potential at later passages [113,114].	Faster proliferation than BM-MSCs; good expansion capacity, but lower telomerase activity than UDSCs [113,114].
Core MSC Markers (ISCT)	Positive for CD73, CD90, CD105, CD44. Negative for CD34, CD45, HLA-DR [3,4,5,39].	Positive for CD73, CD90, CD105. Negative for CD34, CD45 [49].	Positive for CD73, CD90, CD105. Negative for CD34, CD45 [49].
Renal Progenitor Markers	Expresses CD24, CD133, CD106, CD224. Expresses nephrogenesis transcription factors (SIX2, CITED1, WT1, PAX2, PAX8) indicating epigenetic priming for renal fate [5,40,41,42,48,52,53,54,55,56,57].	Absent. Do not express renal progenitor markers or SIX2. Lack of intrinsic kidney-specific identity [54].	Absent. Do not express renal progenitor markers or SIX2. Lack of intrinsic kidney-specific identity [2].
Pluripotency Profile	More robust signature than conventional MSCs. Expresses OCT4, SOX2, NANOG, SSEA-4, TRA-1-60, TRA-1-81 [5,47,51].	Generally negative for embryonic/pluripotency markers (SSEA-4, TRA-1-60/81) [49].	Generally negative for embryonic/pluripotency markers [49].
Renal Homing (CD44-HA axis)	Extremely high CD44 expression (>99.5%). Lower NK cell cytotoxicity confers a survival advantage at the injury site [21,25,68,100].	Expresses CD44, but levels are variable and decline with passage, resulting in weaker homing to injured kidney [100,115].	Expresses CD44, but homing efficiency to renal tissue is moderate and inferior to UDSCs [116].
Key Anti-Fibrotic Paracrine Mechanism	Kidney-Specific: Secretes PGE2 to activate endogenous Sox9^+^ renal progenitors via the Yap pathway. Epigenetically aligned; requires no artificial preconditioning [48,79,96,97,98].	General: Relies on generic paracrine secretion (VEGF and HGF). Often requires artificial “preconditioning” (hypoxia, gene editing) to enhance renal efficacy [117].	General: Relies on generic paracrine secretion. Like BM-MSCs, it lacks specific pathways to activate intrinsic renal progenitors [117].
Extracellular Vesicle (EV) Cargo	EVs carry high levels of Klotho (anti-aging/anti-fibrotic), miR-216a-5p (targets PTEN/Akt), and miR-146a-5p (targets IRAK1/NF-κB) [69,70,73,74,75]	EVs carry various miRNAs but lack intrinsic Klotho expression [118].	EVs carry pro-angiogenic miRNAs but lack intrinsic Klotho expression [119].
Anatomical Precision in CKD	High precision: CD106^−^ subsets target proximal tubules (tubulointerstitial fibrosis); CD106^+^ subsets target Bowman’s capsule (glomerulosclerosis) [8,20]	Low precision: General homing to inflamed areas without specific targeting to distinct nephron compartments.	Low precision: General homing to inflamed areas without specific targeting to distinct nephron compartments.

## Data Availability

No new data were created or analyzed in this study. Data sharing is not applicable to this article.

## Abbreviations

The following abbreviations are used in this manuscript:

UDSC	Urine-Derived Stem Cell
MSC	Mesenchymal Stem Cell
AD-MSC	Adipose-Derived Mesenchymal Stem Cell
CKD	Chronic Kidney Disease
ECM	Extracellular Matrix
SOX2	Sex Determining Region Y-Box 2
OCT4	Octamer-Binding Transcription Factor 4
KLF4	Kruppel-Like Factor 4
c-MYC	MYC Proto-Oncogene
iPSC	Induced Pluripotent Stem Cell
SIX2	SIX Homeobox 2
CITED1	CBP/P300-Interacting Transactivator with Glu/Asp-Rich Carboxy-Terminal Domain 1
WT1	Wilms Tumor 1
PAX2	Paired Box Gene 2
PAX8	Paired Box Gene 8
UMOD	Uromodulin
SLC12A1	Solute Carrier Family 12 Member 1
NR3C2	Nuclear Receptor Subfamily 3 Group C Member 2
NPHS1	Nephrin
CSPG4	Chondroitin Sulfate Proteoglycan 4
CDH1	Cadherin 1 (E-cadherin)
vWF	von Willebrand Factor
eNOS	Endothelial Nitric Oxide Synthase
VEGF	Vascular Endothelial Growth Factor
KDR/VEGFR-2	Kinase Insert Domain Receptor
FLT-1/VEGFR-1	Fms-like Tyrosine Kinase-1
PDGF	Platelet-Derived Growth Factor
FGF	Fibroblast Growth Factor
HGF	Hepatocyte Growth Factor
SIX2^+^	SIX2-Positive Nephron Progenitor Cell
TGFβ-SMAD2/3	Transforming Growth Factor Beta-SMAD Family Member 2/3 Signaling Pathway
sEVs	Small Extracellular Vesicles
IL-1	Interleukin-1
IL-8	Interleukin-8
TGF-β	Transforming Growth Factor Beta
MCP-1	Monocyte Chemoattractant Protein-1 (CCL2)
GM-CSF	Granulocyte-Macrophage Colony-Stimulating Factor
miRNAs	MicroRNAs
Th1/Th17	T Helper 1/T Helper 17 Cell
TNF-α	Tumor Necrosis Factor Alpha
IL-6	Interleukin-6
CTGF	Connective Tissue Growth Factor
GFR	Glomerular Filtration Rate
CD44	Cluster of Differentiation 44
BMSC	Bone Marrow-Derived Mesenchymal Stem Cell
